# Meta-Inflammation in Type 2 Diabetes Mellitus: Theoretical, Methodological, and Clinical Aspects

**DOI:** 10.3390/ijms27198891

**Published:** 2026-10-07

**Authors:** Yulia Zhuravleva, Evgenii Gusev

**Affiliations:** Institute of Immunology and Physiology, Ural Branch of the Russian Academy of Sciences, 620078 Ekaterinburg, Russia; gusev36@mail.ru

**Keywords:** type 2 diabetes mellitus, prediabetes, systemic metabolic inflammation, low-grade systemic inflammation, chronic systemic inflammation, metabolic syndrome

## Abstract

Currently, there is no doubt that insulin resistance (IR) in prediabetes and type 2 diabetes mellitus (T2DM) is associated with chronic systemic metabolic inflammation (SMI). SMI is typically considered a form of low-grade systemic inflammation (LGSI). At the same time, one cannot rule out the possibility of the transformation of LGSI into more pronounced forms of chronic systemic inflammation (ChSI), typical of systemic autoimmune diseases and characterized by a high severity of systemic inflammatory response (SIR). The aim of this research is to assess the pathogenetic role of SMI as a general pathological process in the pathogenesis of prediabetes and T2DM, as well as differentiating SMI into LGSI and ChSI. The study included two main groups—T2DM (*n* = 65) and prediabetes (*n* = 24)—as well as three control groups of healthy individuals of different ages (*n* = 50, *n* = 89, and *n* = 22). In the patients of the main groups, metabolic syndrome (MetS) was diagnosed in accordance with the American Heart Association criteria, and metabolic indices (HOMA-IR, HOMA-islet, and TyG) were calculated. The levels of C-reactive protein (CRP) and cytokines (TNFα, IL-6, IL-8, and IL-10) were determined in blood plasma as SIR parameters, and the levels of cortisol (690 nmol/L or <100 nmol/L), D-dimer (>250 and >500 ng/mL), and troponin I (>0.2 ng/mL) were used as additional SMI criteria. Based on molecular inflammatory markers, the ChSI and LGSI scales were calculated using the authors’ original method. SMI in T2DM and prediabetes manifested itself as both LGSI (in 57% of cases in T2DM and 46% in prediabetes) and ChSI (in 18.5% of cases in T2DM and 8.3% in prediabetes). The verification of SMI based on the frequently used criterion of CRP levels above 3 mg/L turned out to be ineffective in our study (this criterion was detected in the control groups in 24–45%). Therefore, we used CRP > 10 mg/L in combination with cytokines as the SIR criterion. Additionally, we identified the SMI criteria: D-dimer > 250 ng/mL in 27.7% of T2DM and 0% in the control groups and prediabetes; cortisol 15.4% in T2DM and 8.3% in prediabetes; and troponin I 7.7% in T2DM and 4.2% in prediabetes. SMI (LGSI and ChSI) develops in the majority of patients with prediabetes (54.1%) and T2DM (75.4%). To assess SMI, it is advisable to use the integral SIR criteria in combination with the markers of chronic stress, tissue damage, and coagulopathy.

## 1. Introduction

Type 2 diabetes mellitus (T2DM) is a chronic disease caused by complex interactions of genetic, biological, behavioral, and social factors and is characterized by a progressive loss of insulin secretion in pancreatic islet beta cells, which typically occurs following the onset of insulin resistance (IR) and metabolic syndrome (MetS) [1,2,3]. The epidemiology of T2DM has changed, largely due to rising rates of obesity and metabolic disorders, including among young individuals [4,5]. Chronic hyperglycemia and hyperlipidemia with IR and T2DM lead to glucotoxicity and lipotoxicity effects, which can cause damage to various organs and subsequently lead to vascular and other complications: retinopathy, nephropathy, neuropathy, hypertension, ischemic heart disease, thrombophilia, accelerated development of atherosclerosis, and chronic heart failure [6,7]. In the pathogenesis of MetS and T2DM, there is a close relationship between IR and the systemic phenomenon of low-grade systemic inflammation (LGSI), which in this context is defined as systemic meta-inflammation (SMI) [8]. In turn, IR and other chronic homeostatic disturbances (persistent allostasis) are characteristic of tissue aging and are associated with the activation of the innate immune system and other manifestations of pro-inflammatory tissue stress (inflamm-aging) [9]. In this case, inflamm-aging in elderly patients can be considered one of the components of SMI pathogenesis. At the same time, a number of authors view SMI as one of the pathogenetic components of MetS, as well as a plasma/serum C-reactive protein (CRP) level of ≥3 mg/L as an addition to the five main criteria for MetS [10,11,12,13]. According to the results of a meta-analysis, a CRP value of ≥3 mg/L and elevated IL-6 levels can be considered risk factors for vascular complications and chronic heart failure [14,15]. At the same time, other studies have noted that there is insufficient evidence to support the use of high-sensitivity CRP testing for predicting complications of cardiovascular disease [16]. In addition, many authors note that a CRP range of 3–10 mg/L falls within the reference range for physiological values (<10 mg/L) and can serve as a prognostic factor only in the presence of additional risk factors for cardiovascular diseases [17,18,19,20]. We also confirm that a plasma CRP level of ≥10 mg/L—but not lower concentrations of CRP—is a specific criterion for verifying inflammatory processes of various origins and that at least 98% of healthy individuals aged 18–55 have CRP levels below 10 mg/L [21,22]. However, what does “inflammation” mean in this context?

From the standpoint of general pathology, inflammation can be subdivided into canonical inflammation—characterized by a localized focus of inflammation and, in some cases, a systemic inflammatory response (SIR) that is typically protective in nature—and systemic inflammation. Canonical inflammation is generally a protective process, but it can take on a dysfunctional and maladaptive character when genetically determined cellular stress and inflammation programs are damaged or ineffective, for example, in autoimmune, allergic, and autoinflammatory diseases, including through transformation into other forms of general pathological inflammatory processes [21,23]. In turn, systemic inflammation (SI), which is based on systemic microcirculatory disorders, is generally a disadaptation process [24].

The main phenomena of SI are the intravascular activation of pro-inflammatory plasma and cellular blood factors and the systemic activation of endothelial cells and macrophages of the reticuloendothelial system, which together lead to intravascular microthrombosis, systemic “inflammatory microcirculation,” and clinically significant or latent microcirculatory disturbances. For example, acute SI (sepsis, trauma, massive obstetric hemorrhage, and stroke) is characterized by a cytokine storm and life-threatening resuscitation syndromes, including refractory shock, disseminated intravascular coagulation (DIC), and acute respiratory distress syndrome [21,25,26]. Chronic SI (ChSI), in turn, is usually characterized by latent microcirculatory disorders. In keeping with the above, we propose the following definition: “Systemic inflammation is a general multi-syndromic, phase-specific or stadium-specific (in the case of the chronic variant) pathological process evolving in systemic injury and characterized by total inflammatory reactivity of endotheliocytes, plasma and blood cell factors, connective tissue, and, at the terminal stage, microcirculatory disorders in vital organs and tissues” [24]. Within the framework of this definition, LGSI can be considered as an early, marginal stage of ChSI, or pre ChSI, which is characterized by moderate manifestations of SIR and endotheliosis, serving nonetheless as the pathophysiological basis for many diseases, including hypertension and atherosclerosis [27]. From this standpoint, it is necessary to take into account that within the framework of SMI, the systemic inflammatory process in some patients can transform from LGSI (pre-ChSI) into ChSI itself with more pronounced inflammatory criteria. Moreover, in some cases, cytokinemia in ChSI can take on the character of a cytokine storm, which is typical for acute critical conditions in patients with sepsis and acute trauma [24]. We define this condition as hyper-ChSI. Thus, SMI is a heterogeneous process, and it is advisable to differentiate it into LGSI/pre-ChSI, ChSI itself, and hyper-ChSI in some patients.

Cellular and tissue stress is the common basis of various pathological and even extreme physiological processes [28,29,30]. In this case, the implementation of a pro-inflammatory receptor and secretory phenotype by many cells leads to tissue stress and, in some cases, an SIR. In this regard, the SIR may be a manifestation of not only pathology but also extreme physiology, a risk factor for pathology. For example, strenuous muscle activity can lead to an SIR and, subsequently, to systemic alterations and organ dysfunction [31,32]. This fact makes it necessary to differentiate SI—particularly its marginal form of LGSI—from SIR as a systemic manifestation of canonical inflammation and other causes that are functionally distinct from SI [24].

The causal relationship between IR, MetS, and T2DM, on the one hand, and SMI, on the other, takes the form of a vicious pathogenic cycle. When studying the mechanisms underlying this relationship, one comes across a number of unresolved issues. However, comprehensive conceptual frameworks are emerging that systematize this relationship [33,34,35,36,37]. These ideas can be summarized as follows: (1) Tissue aging and metabolic dysfunction lead to pro-inflammatory activation of stromal macrophages and low-grade inflammation in insulin-dependent tissues (liver, skeletal muscle, and adipose tissue) and in pancreatic islets. (2) At the cellular level, insulin receptor signaling pathways in various cells are closely linked to pro-inflammatory cellular stress signaling pathways. This interaction leads to the impaired function of GLUT4, the primary transmembrane glucose transporter in adipocytes and skeletal muscle, as well as to abnormalities in glucose metabolism, the activation of gluconeogenesis, and the impaired formation of lipid transport forms in hepatocytes. (3) Insufficient compensatory insulin production by beta cells, an imbalance in metabolic cycles, and disruptions in the production and blood levels of adipokines, myokines, and hepatokines—which contain regulatory RNAs in extracellular vesicles—contribute to endotheliosis and other SMI-related phenomena [38]. (4) At the same time, SMI in insulin-dependent tissues may clinically manifest as metabolically associated steatosis liver disease (MASLD), formerly known as nonalcoholic fatty liver disease (NAFLD), sarcopenia and morbid obesity (an increase in the number and pro-inflammatory activation of macrophages and insulin-resistant adipocytes in fat depots), and pathological accumulation of fatty acids and atherogenic lipoproteins in the blood [39,40]. (5) The progression of SMI and metabolic dysfunction causes various pathologies of the cardiovascular system and other organs, along with the formation of numerous feedback loops that contribute to the severity of T2DM. (6) A separate aspect of this issue involves IR- and SMI-related disturbances in the microbiome and intestinal barrier function [41]. (7) In general, SMI is an aberrant, a priori maladaptive form of inflammation that is unable to localize and effectively eliminate the factors causing tissue alteration but is capable of further exacerbating them.

In T2DM, a qualitative transformation of the inflammatory process is possible, for example, from local chronic low-grade inflammation to canonical inflammation. This process is observed during the progression of hepatic steatosis (hepatosis) to steatohepatitis or the progression of diabetic kidney disease from local manifestations of LGSI to fibrinous inflammation, which is one variant of canonical inflammation [42,43,44]. In turn, at the systemic level, SMI in T2DM may, in some cases, go beyond LGSI and progress to the ChSI stage and even to hyper-ChSI [22].

It is necessary to take into account that certain infections and their long-term consequences—including HIV, pulmonary tuberculosis, periodontitis, and long COVID—may also contribute to the development of LGSI and, through this mechanism, exacerbate IR and create a complex inter-relationship with T2DM [45,46,47,48]. IR and T2DM, in turn, may have a negative impact on diseases associated with local clinical manifestations of LGSI, such as osteoarthritis and neurodegenerative diseases [49,50]. With this in mind, it is not surprising that T2DM is associated with various neuropsychiatric disorders [51,52].

A separate issue in this context is the relationship between SI and chronic stress of the neuroendocrine system, in particular, dysfunction of the hypothalamic–pituitary–adrenal (HPA) axis [53,54,55]. First, stress/distress in the HPA axis is one of the SI phenomena that can be assessed using plasma cortisol levels [56,57,58]. Second, neuroendocrine stress responses play a significant role in the pathogenesis of IR and T2DM [59,60]. Third, even chronic psychogenic stress can trigger low-grade neuroinflammation in certain brain structures due to neurotransmitter imbalance, with the associated dysfunction of the HPA axis and the sympathetic nervous system contributing to the generalization of inflammation in the form of systemic LGSI [61,62,63]. All of these determine the necessity of taking into account the stress response of the HPA axis when assessing SMI.

In view of the above, the aim of our research is to assess the pathogenetic role of SMI as a general pathological process in the pathogenesis of prediabetes (ICD-11, codes 5A40.0 and 5A40.1) and T2DM (ICD-11, code 5A11), as well as differentiating SMI into LGSI, ChSI, and, in some patients, the detection of hyper-ChSI. More specific objectives included determining the association of certain indicators and integral criteria for SMI/LGSI, SMI/ChSI, and hyper-SMI with a previous history of COVID-19 (ICD-11, code QA42), the presence of MetS (ICD-11, code 5A44), and indices of obesity and insulin resistance (HOMA-IR, HOMA-islet, and TyG). In this study, the SMI parameters used were the plasma levels of CRP, cytokines (TNF-α, IL-6, IL-8, and IL-10), and procalcitonin (PCT) as indicators of the systemic inflammatory response (SIR), as well as cortisol (a marker of chronic stress), D-dimer (a marker of DIC), and myoglobin and myocardial-specific isoforms of troponin I (TnI) as markers of tissue damage. The integral indexes were utilized to separately verify SMI associated with LGSI (SMI/LGSI) and SMI associated with ChSI (SMI/ChSI), as were the six qualitative levels of SIR (on a 0–5-point scale) [21,64]. Additional screening indicators for SIR were based on routine hemogram parameters: various ratios of neutrophils, lymphocytes, monocytes, and platelets (NLR, PLR, and SII). The study also took into account standard criteria for comorbid conditions.

## 2. Results

### 2.1. General Characteristics of the Two IR Groups (T2DM and Prediabetes)

An analysis of the basic clinical characteristics of the groups of patients with T2DM and prediabetes showed that they were comparable in terms of age, sex, and most comorbid somatic diseases, with the exception of cardiovascular diseases (Table 1). Most of the patients in both groups had signs of obesity and dyslipoproteinemia. All patients with T2DM and prediabetes met the criteria of IR, as verified by at least two out of the three IR scales (HOMA-IR, HOMA-islet, and TyG). The scores for these criteria as per the IR scales in the T2DM and prediabetes groups were found as expected. According to the HOMA-IR scale, the groups are comparable (*p* > 0.05); the TyG values are higher in T2DM, while the HOMA-islet values, conversely, are higher in prediabetes (*p* < 0.05), which indicates greater IR and worse islet function in T2DM.

### 2.2. Assessment of SMI Criteria

#### 2.2.1. Preliminary Remarks on the Assessment of SMI Integral Indices

Three scales were used to verify SMI: (1) the RL scale—for a comprehensive assessment of SIR; (2) the ChSI scale—to verify SMI/ChSI; and (3) the LGSI scale—to verify SMI/LGSI. In addition, some patients were considered to have hyper-ChSI based on their RL ≥ 4.

To establish the minimum reactivity level required for SIR verification, namely RL-1, at least two indicators (out of the five on the RL scale) must slightly exceed the upper limit of the reference range (up to twice the upper limit), or one indicator must show a more significant change.

In most cases, SMI/ChSI (a 3-point score or higher on the ChSI scale) is diagnosed by the presence of RL-3, which is atypical not only for chronic but also for acute canonical inflammation. The same is diagnosed when RL-2 is present along with an additional criterion, which is typically a D-dimer level above 500 ng/mL. Within the SMI/ChSI framework, we should specifically highlight hyper-ChSI, as it is associated with RL-4, i.e., an SIR level characteristic of the cytokine storm phase of acute, life-threatening SI [21].

SMI/LGSI confirmation (2 points on the LGSI scale) is possible only in the presence of metabolic dysfunction (IR) and a minimum SIR level (RL-1). In the absence of an SIR (UR-0) but with IR present, SMI/LGSI can also be confirmed, but only in three cases, namely (1) CRP levels below the SIR criteria (3–10 mg/L) + at least one cytokine on the SIR scale (IL-6, IL-8, IL-10, and TNF-α) exceeding the normal range (N); (2) C-reactive protein 3–10 mg/L + D-dimer > N (>250 ng/mL); and (3) CRP > N (>10 mg/L). Thus, the LGSI in the SMI model can be determined even when RL-0 is present, which in other cases (in the absence of IR) indicates the absence of an SIR. In turn, the presence of LGSI (pre-ChSI) in the “inflamm-aging” subtype (without IR criteria) is determined only when RL-1 or RL-2 is present without the ChSI criterion (ChSI score ≤ 2 points).

#### 2.2.2. Evaluation of Separate and Integral SMI Parameters

Based on the above and the data in Table 2, SMI in our study can be divided into two categories, namely (1) SMI/LGSI (in 57% of cases with T2DM and in 46% of cases with prediabetes) and (2) SMI/ChSI (in 18.5% of cases with T2DM and in 8.3% of cases with prediabetes), including hyper-ChSI (in 1.5% of cases with T2DM and in 4.2% of cases with prediabetes). In both cases, these values differed qualitatively from the control (0%) but did not differ significantly from one another (*p* > 0.05).

CRP (especially at concentrations of 3–10 mg/L), as well as PCT and IL-10 when used as SIR criteria, did not demonstrate high diagnostic efficacy in distinguishing between the two IR groups from the control. In contrast, in T2DM and prediabetes, TNF-α (which exceeded N in 53.8% and 37.5% of cases, respectively) and IL-8 (which exceeded N in 58.5% and 45.8% of cases, respectively) made the greatest contribution to the verification of SIR (RL ≥ 1). At the same time, the median values for IL-10, CRP, PCT, IL-6, and the ChSI scale scores were significantly (*p* < 0.05) higher in patients with T2DM compared with those with prediabetes.

A comparative analysis of the immune–inflammatory indices across the groups (Table 3) revealed a mixed picture: the SII and NLR indices were comparable between patients with T2DM and the control group, while the PLR index was even significantly higher in the control group. This fact makes it questionable whether this indicator can be used to assess the inflammatory status of patients when assessing meta-inflammation.

IL-8 had the greatest impact on the development of SIR in inflamm-aging without clinical manifestations of IR. That said, the SMI and inflamm-aging groups differed in several indicators. Thus, SMI was characterized by higher ChSI scores (in % and in absolute values), a higher frequency (in %) of pronounced SIR manifestations (RL ≥ 2), elevated levels of D-dimer and TNF-α, but a lower proportion (in %) of RL-0 (Table 4). Thus, inflamm-aging influences (RL-1 in 18.2% of cases) but does not fully determine the development of SMI. In this context (SMI and/or inflamm-aging), TNF-α primarily indicates the pro-inflammatory function of tissue macrophages (including vascular macrophages), while IL-8 may be produced in high quantities by activated endothelial cells [65,66,67].

The ChSI scale criteria, which reflect the presence of tissue damage (TnI > 0.2 ng/mL and/or myoglobin > 60 ng/mL) and HPA axis dysfunction (cortisol > 690 or <100 nmol/L), were identified only in a relatively small number of patients, predominantly those with T2DM. This is both a general characteristic of ChSI and its distinction from acute SI, in which these criteria are more frequent and more pronounced [21]. Meanwhile, these criteria make it possible to verify and clarify the pathogenetic characteristics of ChSI in some patients.

A distinctive feature of T2DM is D-dimer levels that exceed the upper limit of normal in 27.7% of cases (>250 ng/mL, LGSI criterion) and, in some patients (10.8%), meet the ChSI scale criterion (>500 ng/mL). We also note that D-dimer concentrations in the range of 100–250 ng/mL can be considered either an extreme normal value (5.6% in the control group) or a risk factor for intravascular coagulopathy in T2DM (40%) and prediabetes (33%).

#### 2.2.3. The Impact of a History of COVID-19 and the Presence of MetS Criteria on SMI Parameters

A past COVID-19 infection in a patient’s medical history did not have a significant impact on the development of SMI in the integrated IR group (T2DM + prediabetes), except for a statistically significant increase in serum IL-6 levels and a decrease in the frequency of D-dimer levels >500 ng/mL (Supplement, Table S1). However, we were surprised to find that the presence of MetS in patients did not have a significant effect on the SMI criteria, except for a statistically significant increase in LGSI scores (Supplement, Table S2). Probably, when verifying MetS, obesity can be considered as one of the criteria for metabolic dysfunction rather than as a mandatory (attribute-based) criterion. Perhaps, for the verification of MetS, the criteria of IR scales (metabolic indices) should also be taken into account. Moreover, in some cases, consideration should be given not only to the volume of adipose tissue but also to the clinical and laboratory criteria for morbid obesity, including the measurement of pro-inflammatory lipokines in the blood and certain stress-related non-coding RNAs from the adipose tissue and the liver, as well as other molecular markers of IR [35].

#### 2.2.4. Correlation and ROC Analyses of SMI Criteria and Metabolic Dysfunction Markers

A past COVID-19 infection in a patient’s medical history did not have a significant influence. Correlation analysis demonstrated a clear division of the studied parameters into two clusters: “inflammation” and “metabolism” (Supplement, Table S3). As is well known, this method does not enable one to establish pathogenetic cause-and-effect relationships, but it does reflect the degree of synchrony among the parameters being measured. Thus, in the groups we studied, it would not be effective to assess inflammation levels based on changes in metabolic parameters and vice versa. As expected, the RL scale, the ChSI scale, TNF-α, and IL-8 correlated most strongly with one another in the “inflammation” cluster (r > 0.8); however, the correlations within the “metabolism” cluster are less significant, which suggests that it may be appropriate in some cases to use several alternative criteria to verify IR.

The results of the ROC analysis presented in Table 5 confirm that the RL scale (AUC > 0.7, *p* < 0.05) and the ChSI scale (AUC > 0.8) are capable of differentiating patients with IR from the control group well, as are D-dimer (AUC ≥ 0.9), IL-8 (AUC ≥ 0.9), and TNF-α (AUC ≥ 0.7). However, only D-dimer is effective (AUC ≥ 0.7, *p* < 0.05) in separating T2DM from prediabetes (Table 6). Among the IR scales, only the HOMA-islet scale is able to effectively (AUC ≥ 0.7, *p* < 0.05) distinguish between T2DM and prediabetes. All values of 0.6 ≤ AUC < 0.7 were classified as “satisfactory”; 0.7 ≤ AUC < 0.8 as “good”; 0.8 ≤ AUC < 0.9 as “very good”; and AUC ≥ 0.9 as “excellent” (*p* < 0.05).

## 3. Discussion

Both prediabetes and T2DM are characterized by the presence of IR criteria in 100% of cases. At the same time, IR-related conditions are also characterized by the development of SMI: prediabetes in 54.1% of cases and T2DM in 75.4% of cases. In both instances, the borderline stage associated with LGSI (SMI/LGSI) predominates in 45.8% of cases with prediabetes and in 56.9% of cases with T2DM, while the high-intensity stage—SMI/ChSI—is detected in 8.3% of cases with prediabetes and in 18.5% of cases with T2DM. However, these differences in SMI between prediabetes and T2DM are not statistically significant (*p* > 0.05) and are observed in patients with prediabetes and T2DM regardless of whether they are obese or not. Meanwhile, in patients with T2DM, elevated plasma D-dimer levels (>250 ng/mL) are observed in 27.7% of cases, whereas they are not detected in patients with prediabetes (*p* < 0.05). Other studies have also showed a link between elevated D-dimer values and cardiovascular diseases, obesity, and T2DM [68,69,70]. All of this argues for the inclusion of blood D-dimer levels in the integral criteria not only for SMI/LGSI but also for other variants of LGSI at concentrations below the DIC threshold (>500 ng/mL). Moreover, in patients with T2DM, a stress/distress response of the hypothalamic–pituitary–adrenal (HPA) axis is observed in 15.4% of cases as an additional criterion on the ChSI scale (elevated or reduced cortisol levels). Tissue alteration criteria, on the other hand, are even less common. Other researchers have also identified HPA axis dysfunction in IR and T2DM [71,72]. The widely used LGSI marker CRP has not demonstrated high diagnostic significance as an independent criterion, so we recommend using it for the diagnosis of LGSI only in the presence of insulin resistance and in combination with other markers.

**Figure 1:**
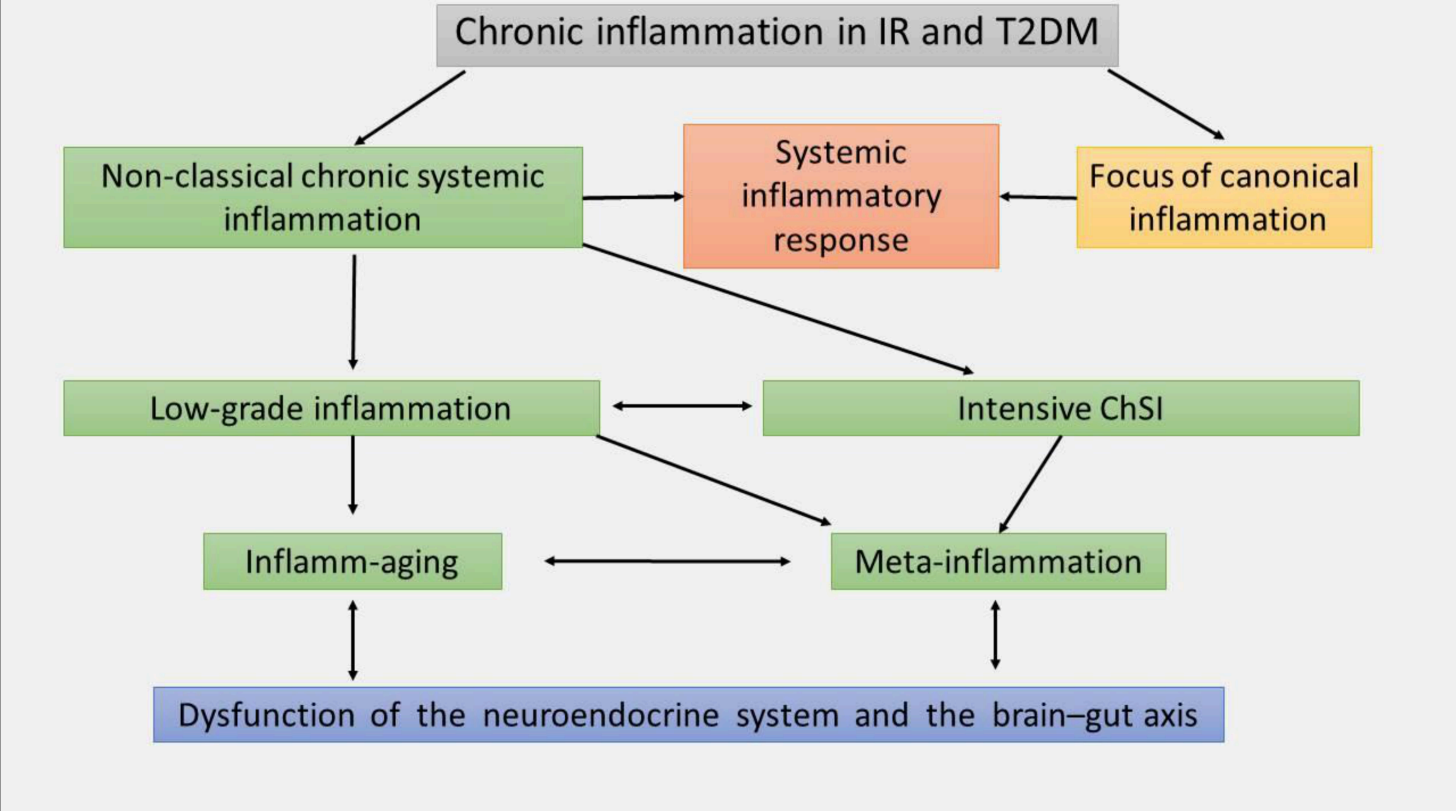
Conceptual scheme of the components and interactions of meta-inflammation as a general pathological process.

Because the aging process itself influences the manifestations of systemic inflammation, age is a potential confounding factor in the assessment of this pathological process. Therefore, the inclusion of a comparison group consisting of older individuals was partly motivated by the need to take this aspect into account. The comparison group—patients aged 61–83 (an age range comparable to that of the prediabetes and T2DM groups)—who did not have clinically significant signs of IR (did not meet the criteria for prediabetes or T2DM), showed signs of SIR (RL ≥ 1) in 18.3% of cases but not of ChSI (0%). These signs can be interpreted as indicative of LGSI associated with tissue aging. Thus, both the metabolic mechanisms of IR and inflamm-aging may play a role in the development of SMI. At the same time, the dysfunction of the gut–brain axis may contribute to the development of both SMI and inflamm-aging, leading to the formation of a vicious pathogenic cycle [73,74,75,76]. In both cases, it may be necessary to differentiate SIR where there are pronounced foci of canonical inflammation and other causes of SIR that are independent of LGSI and ChSI (Figure 1).

First, consider this differentiation from the perspective of the evolutionary aspects of inflammation as a general biological process. SIR phenomena (the accumulation of soluble factors and innate immune cells in the hemolymph), along with localized anti-inflammatory reactions from phagocytes in response to tissue damage, have already been identified in various types of invertebrates [77,78]. Vertebrates have developed a secondary cellular lining that separates the blood from other tissues, called the endothelium [79]. This has established that a full-fledged canonical inflammatory response—including an exudative –vascular reaction and the selective migration of leukocytes to the site of inflammation—is possible only in vertebrates, while the presence of shock-inducing microcirculatory disorders is characteristic of mammals and, to a limited extent, of birds [80]. The phenomenon of “inflammatory systemic microcirculation” in mammals is determined primarily not only by homeothermy but also by the high degree of organization of the complement system, the hemostatic system, the kallikrein–kinin system, the endothelium of the microvascular network, and the immunoneuroendocrine system as a whole [80,81]. Thus, SIR may be associated with evolutionarily different forms of inflammation.

Microcirculatory dysfunction in SMI, as well as in other chronic forms of SI, predominantly manifests itself in a latent form as endotheliosis. However, in T2DM, morphofunctional abnormalities are observed in various components of the endothelial layer of different blood vessels, including endothelial cells, pericytes, and macrophages of the reticuloendothelial system [82,83,84,85]. These changes are associated with oxidative stress, damage to the endothelial glycocalyx, and other manifestations of cellular and tissue pro-inflammatory stress. Characteristic features of endotheliosis derive from the unique role of scavenger receptors on endothelial cells and vascular macrophages that lie at the intersection of metabolism, immunity, and inflammation. The same applies to increased permeability of the arterial endothelium to atherogenic lipoproteins, activation of the renin–angiotensin system, and overproduction of endothelin I. Against the backdrop of impaired nitric oxide bioavailability (inhibition of constitutive endothelial NO synthase), these factors contribute to hypertension, atherosclerosis, thrombophilia, and impaired microvascular function in various organs [27,35].

Thus, endotheliosis in SMI is generalized in nature and contributes to pathological changes in various compartments of the vascular network, including the microcirculatory zone. Here, the term “microcirculation” refers to blood flow within microcirculatory units, including arterioles, capillaries, and postcapillary venules [86,87,88]. Diabetic microangiopathy encompasses retinopathy, nephropathy, and neuropathy, as well as a typical decrease in capillary network density and dysfunctional microvascular responses in other tissues in T2DM [89,90,91,92,93]. Interestingly, atherosclerotic changes in the arteries and microvascular dysfunction in T2DM and MetS correlate with one another, and these abnormalities can be described as accelerated vascular aging [94]. Also of interest are data suggesting a possible reverse effect, namely endotheliosis as a risk factor for the development of IR and T2DM [95].

Since SMI is not the only variant of ChSI, it is interesting to compare the probability of developing SIR, ChSI, and hyper-ChSI (RL ≥ 4) as per the standard RL and ChSI scales in cases of T2DM and prediabetes, as well as in other chronic diseases we have previously studied [21,47] (Table 7).

The first thing to note is that T2DM is in cluster 3 (10–50% ChSI) along with four systemic autoimmune diseases. A significantly more pronounced role of ChSI in the pathogenesis of the autoimmune process is observed in systemic lupus erythematosus (SLE, group No. 19). At the same time, in SLE, the presence of hyper-ChSI is associated not only with RL-4 but, in some patients receiving ambulatory treatment, also with RL-5, which manifests as a thousand- to ten-thousand-fold increase in pro-inflammatory cytokines, a condition that leads to death in most cases of acute SI [21]. This raises two questions. (1) What are the mechanisms that protect against a cytokine storm in ChSI? (2) What levels of hypercytokinemia in ChSI might cause the phenomenon of systemic excitotoxicity? Most likely, these are not the levels typical of LGSI. It is important to note that LGSI is a marginal stage of ChSI that can be differentiated from other causes of SIR only in certain pathological conditions. For example, in group No. 6 (chronic heart failure, ages 70–93), SIR can be linked to LGSI and tissue aging, as well as systemic hypoxia and other secondary changes in homeostasis. The relatively low frequency of ChSI in the N6 group suggests that the development of ChSI—and even more so, hyper-ChSI—in patients with IR (prediabetes and T2DM) cannot be explained solely by aging and cardiovascular disease. In group No. 10, the high probability of SIR development in young men is primarily associated with the presence of a pronounced purulent-inflammatory process of the canonical type (chronic phlegmon involving the skin, subcutaneous fat, and muscles). Systemic immune-mediated diseases (autoimmune and autoinflammatory) are also characterized by the presence of foci of classic inflammation in various organs, including localized angiopathies, which makes the diagnosis of LGSI in this category of pathologies a difficult task. In contrast, HIV, herpesvirus infections, viral hepatitis, latent tuberculosis, and certain other chronic infections that frequently co-occur with T2DM can be characterized from the standpoint of LGSI [96,97,98,99,100]. To verify LGSI in cases of such infections, it is necessary to use additional criteria for endotheliosis. To this aim, we supplemented the LGSI scale with a plasma endothelin-I level above the normal range [47]. Thus, although LGSI can be viewed as a general pathological process, its verification and characterization in various pathologies require a differentiated approach. A specific aspect of the problem under consideration is the possibility of long-term stabilization or even elimination of ChSI. This possibility is supported by the results of treating patients on programmed hemodialysis (group No. 19) through successful kidney transplantation (group No. 4). In addition, recent findings on the treatment of T2DM also indirectly suggest this possibility [101,102,103].

Thus, it is currently impossible to definitively establish a causal link between the development of T2DM and inflammation. Evidence supporting the primacy of inflammation includes the following: (1) T2DM often develops against a background of “inflamm-aging”; (2) many chronic diseases linked to systemic inflammation are risk factors for insulin resistance [35]; and (3) gene polymorphisms associated with pro-inflammatory cellular stress—such as those involving the NLRP3 inflammasome—are risk factors for T2DM [104]. Conversely, our data suggest that metabolic changes precede meta-inflammation; specifically, meta-inflammation is not an intrinsic factor of insulin resistance, and this process appears to develop in the majority—though not all—patients with T2DM.

This study has a number of limitations. First, the limited sample size restricts the assessment of potential risk factors. Second, the one-off examination procedure may have underestimated the actual number of patients with SMI and also did not allow for prospective assessment of the clinical consequences of SMI. Third, the study did not use instrumental methods for assessing microcirculation or tissue stress markers that are more specific to endothelial dysfunction, which prevented us from more accurately describing the possible microcirculatory disturbances in these cases. Fourth, patients who have had COVID-19 should not be directly associated with long COVID. A fifth limitation is that differences in therapy among patient groups with IR could have influenced the study results for individual patients. However, we do not consider this potential influence significant, given the relatively high threshold for the development of LGSI—and especially ChSI—which is not reached by all patients with type 2 diabetes. Among the more general limitations, we note the absence of a widely accepted theory of systemic inflammation as a general pathological process distinct from other manifestations of SIR and, consequently, the lack of uniform, universal methodological approaches to the assessment of various forms of SI.

## 4. Materials and Methods

### 4.1. Patients

The study was performed in accordance with the Declaration of Helsinki of 1975 for Human Research and was approved by the Local Ethics Committee of the Institute of Immunology and Physiology, Ural Branch of the Russian Academy of Sciences (Protocol No. 1/26 dated 27 June 2025). All patients signed a voluntary informed consent form to participate in the study.

The study included 89 patients with hyperglycemia who were examined by an endocrinologist at the “Uralskaya” Clinic (Ekaterinburg).

The following were the criteria for excluding patients from the study: a history of myocardial infarction, acute cerebrovascular accident, and/or cardiac or vascular surgery within the past year; chronic heart failure (CHF) classified as functional class IV on the New York Heart Association (NYHA) scale; and end-stage chronic kidney disease (stage 5 CKD), ketoacidosis, diabetic foot ulcers, viral hepatitis, HIV, tuberculosis, type 1 diabetes, autoimmune, autoinflammatory diseases, tumors, and the presence of acute or exacerbated chronic diseases at the time of the study.

All patients were divided into two groups according to the criteria of the American Diabetes Association (ADA) [105]:

1. The “prediabetes” group included 24 patients (median age: 63 years; male-to-female ratio: 38% to 62%). The diagnosis of “prediabetes” was based on the following criteria: hyperglycemia (fasting venous serum glucose level of 5.6–6.9 mmol/L and/or postprandial serum glucose level of 7.8–11.0 mmol/L) and a glycated hemoglobin (HbA1c) level below 6.5%. These criteria correspond to the following diagnoses: impaired fasting glucose (ICD-11, code 5A40.0) or impaired glucose tolerance (ICD-11, code 5A40.1).
2. The “T2DM” group included 65 patients (median age: 66 years; male-to-female ratio: 55% to 45%) with a confirmed diagnosis of T2DM (ICD-11, code 5A11). The criteria for diagnosing T2DM were fasting venous serum glucose level greater than or equal to 7 mmol/L and/or postprandial serum glycemia level greater than or equal to 11.1 mmol/L, as well as glycated hemoglobin (HbAlc) level greater than or equal to 6.5%.

### 4.2. Control Groups

The study included three control groups of healthy volunteers:

1. Control Group 1: The main control group consisted of 89 blood donors with no acute or systemic chronic diseases (median age: 32 years; male-to-female ratio: 55% to 45%). In this group, inflammatory biomarkers were measured via enzyme-linked immunosorbent assay (ELISA) using a Lazurite automated analyzer (Dynex Technologies Inc., Chantilly, VA, USA). The same method was used to verify SMI in patients with IR.
2. Control Group 2: This group comprised blood donors (*n* = 50, including 26 (52%) men and 24 (48%) women, median age: 33 years) whose molecular biomarkers were analyzed using an Immulite chemiluminescent immunoassay system (Siemens Medical Solutions Diagnostics, Malvern, PA, USA). The results of the inflammatory biomarker analysis in this group were compared with data obtained by ELISA to confirm the comparability of these analytical methods for assessing chronic systemic inflammation.
3. Control Group 3 (comparison group). Since the majority of patients (66 individuals) were over 60 years of age at the time of the study, a comparison group was formed. This comparison group consisted of older adults (aged 60–74) without acute or systemic degenerative diseases, exacerbations of chronic diseases, or other inflammatory conditions (*n* = 22, median age: 68 years, male-to-female ratio: 59% to 41%) who were undergoing routine health checkups. The patients did not have T1DM, T2DM, severe heart failure, or systemic destructive chronic conditions (autoimmune diseases, tuberculosis, hepatitis, liver cirrhosis, cancer, or a history of stroke or myocardial infarction); at the time of the study, they were not suffering from any acute illnesses and were not experiencing any exacerbations of chronic conditions.

A comparative analysis of the control groups is presented in Table 8.

### 4.3. Methods

During the study, sociodemographic and clinical data were collected from medical history records. The following clinical parameters were taken into account: diagnosis, comorbid conditions, complications of diabetes, history of COVID-19, weight, height, blood pressure, hemogram parameters, fasting levels of glucose, HbA1c, C-peptide, and lipid panel. Body mass index (BMI) was calculated as the weight in kilograms divided by the square of the height in meters (kg/m^2^). A fasting venous blood sample was obtained to measure the concentration of inflammatory biomarkers using the ELISA method.

#### 4.3.1. Diagnosis of Metabolic Syndrome

The following criteria were used to diagnose metabolic syndrome (MetS) [12]: obesity (BMI > 30) (mandatory criterion); elevated blood pressure (systolic ≥ 130 and/or diastolic ≥ 85 mm Hg); elevated fasting glucose ≥ 5.6 mmol/L; elevated triglycerides ≥ 1.7 mmol/L; and reduced HDL-C < 1.03 mmol/L in males and 1.29 mmol/L in females. Metabolic syndrome was verified in a patient based on the presence of one mandatory criterion (obesity) and at least two additional criteria.

#### 4.3.2. Calculation of Metabolic Indices

Insulin resistance was assessed using the following criteria:

- HOMA-IR (CP) = 1.5 + fasting blood glucose (mmol/L) × fasting C-peptide (pmol/L)/2800.

An elevated HOMA-IR (CP) above 3.5 is a criterion for insulin resistance [106].

- TyG = ln (fasting triglycerides (mg/dL) × fasting glucose (mg/dL)/2).

According to [107], the best TyG index for diagnosing insulin resistance was found to be 4.65, which demonstrated the highest sensitivity (84.0%) and specificity (45.0%). Therefore, a TyG value of 4.65 was selected as the cut-off point.

Islet cell function was assessed using the HOMA-islet index: HOMA-islet = 0.27 × fasting C-peptide (pmol/L)/(fasting blood glucose (mmol/L) − 3.5) + 50.

Low HOMA-islet index values indicate β-cell dysfunction. In the absence of reference values, we used the cut-off values reported by other authors. In [106], the cut-off value for HOMA-islet was found to be 167.58.

#### 4.3.3. Calculation of Complex Immune–Inflammatory Indices

Based on the hemogram parameters, the immuno-inflammatory indices were calculated using the following formulas:

- SII (systemic immune–inflammation index) = (neutrophil count × platelet count)/lymphocyte count;
- NLR (neutrophil-to-lymphocyte ratio) = neutrophil count/lymphocyte count;
- PLR (platelet-to-lymphocyte ratio) = platelet count/lymphocyte count.

The control group for the calculation of immuno-inflammatory indices was formed from healthy volunteers (*n* = 22). The small size of this group did not allow us to determine reference values for the calculated indices; therefore, we relied on the cut-off values reported in the medical literature: 660 for SII [108], 4 for NLR [109], and 165 for PLR [110].

#### 4.3.4. Measurement of Inflammatory Mediator Levels

We studied blood plasma stabilized by citrate, previously frozen at –20 °C. The levels of interleukins-6, -8, and -10, TNFα, CRP, cortisol, myoglobin, troponin I, procalcitonin, and D-dimers in blood plasma samples were analyzed by enzyme immunoassay on a Lazurite automated analyzer (Dynex Technologies Inc., Chantilly, VA, USA).

#### 4.3.5. Assessment of Chronic Systemic Inflammation and LGSI

Molecular inflammatory markers were utilized to calculate the ChSI scale that we had previously developed and tested in patients with various chronic diseases (15 nosologies) [21]. This scale involves the evaluation of the main phenomena of ChSI: SIR, HPA axis dysfunction, intravascular microthrombosis, and systemic alterations. Each of these phenomena was verified using specific criteria (Table 9). The SIR was assessed using a method that included measuring the levels of interleukins: IL-6, IL-8, and IL-10, as well as TNF-α and CRP; based on these values, an integral index of SIR—the reactivity level (RL)—was calculated [21]. The RL calculation method is presented in Supplement, Table S4. The interpretation of reactivity levels is presented in Supplement, Table S5. The RL can have a value from 0 to 5 points. The presence of the other phenomena (based on at least one criterion) was scored as 1 point on the ChSI scale. The points were then summarized, and patients with a total score of 3 or more were diagnosed with ChSI.

For patients in whom the ChSI had been ruled out, LGSI was verified using the original method that involved, in addition to the commonly used SMI criterion (CRP values 3–10 mg/L), assessment of other manifestations of LGSI (Table 10).

The validity of the RL and ChSI scales was established through the study of various acute and chronic diseases [21,26,44,47,58,64]. However, the LGSI scale has not yet been tested in other groups of patients with IR than those mentioned in the article, and we have no information that it has been used in studies independent of ours.

#### 4.3.6. Statistical Analyses

Statistical analyses were performed using the Statistica 12.0 program (Stat Soft, Inc., St., Tulsa, OK, USA) and SPSS for Windows, Version 15.0 (SPSS Inc., Chicago, IL, USA). Shapiro–Wilk tests were used to check the hypothesis that the distribution of samples was not normal. Departure of most parameters from normality, as well as the small number of participants in some groups, determined the use of nonparametric statistical methods. The descriptive statistics are presented by their main characteristics: Me = median; Q1–Q3 = interquartile range. Categorical variables were presented as a percentage (%). We used the Spearman correlation coefficient (r), a non-parametric analytical method. Comparisons between the groups were performed using a Mann–Whitney U-test for continuous variables. The Pearson Chi-square test was utilized to analyze categorical data. The areas under the receiver operating characteristic curve (AUC) were used to evaluate the ability of the biomarkers, RL, and SI scale to discriminate patients with hyperglycemia from control group. The ratio of the AUC and diagnostic significance intervals were rated as follows: 0.9–1.0 = “excellent”, 0.8–0.9 = “very good”, 0.7–0.8 = “good”, 0.6–0.7 = “medium”, and 0.5–0.6 = “unsatisfactory” [111]. All results were considered statistically significant if the *p*-value was <0.05.

## 5. Conclusions

1. Insulin resistance in T2DM and PD is characterized by a high probability of developing systemic meta-inflammation of low and, in some cases, high intensity.
2. To assess meta-inflammation, it is advisable to use comprehensive criteria for the systemic inflammatory response, as well as additional criteria for the HPA axis stress response, tissue damage, and coagulopathy.
3. Increasing intravascular coagulation (elevated D-dimer levels) plays a significant role in the progression of meta-inflammation.
4. The onset of meta-inflammation in insulin resistance may occur independently of obesity, but in older patients, it is linked to inflamm-aging.
5. When diagnosing meta-inflammation, it is necessary to consider alternative causes of a systemic inflammatory response associated with comorbid conditions.

## Figures and Tables

**Table 1:** The clinical characteristics of the groups.

Characteristics	Prediabetes (*n* = 24)	T2DM (*n* = 65)	*p*-Level (Chi-Square)
Cardiovascular diseases	91.7%	100%	**0.02**
Coronary heart disease	16.7%	47.7%	**0.** **0** **0** **8**
Essential hypertension, stage II	41.7%	32.3%	0.41
Essential hypertension, stage III	25%	64.6%	**0.0009**
Chronic heart failure	70.8%	95.4%	**0.** **001**
Among them, chronic heart failure, NYHA classes II- III	20.8%	33.8%	0.23
Chronic heart failure, NYHA class	0	3.1%	0.39
Respiratory diseases	8.3%	10.8%	0.74
Chronic obstructive pulmonary disease	0	1.5%	0.54
Bronchial asthma	8.3%	9.2%	0.89
Gastrointestinal diseases	54.2%	64.6%	0.37
Stomach and duodenum diseases	25%	53.8%	**0.0** **2**
Non-alcoholic fatty liver disease, hepatic steatosis, and non-alcoholic steatohepatitis	33.3%	23.1%	0.33
Chronic pancreatitis, chronic cholecystitis, and cholelithiasis	37.5%	35.4%	0.85
Urinary tract disorders	20.8%	21.5%	0.95
Among them, chronic pyelonephritis	12.5%	6.2%	0.32
Anemia	0	6.2%	0.16
COVID-19 in anamnesis	29.2%	38.5%	0.42
T2DM complications	29.2%	80.0%	**0.0001**
Encephalopathy	29.2%	75.4%	**0.0001**
Diabetic retinopathy	0	3.1%	0.38
Diabetic kidney disease	0	15.4%	**0.0** **4**
With stage 3 chronic kidney disease	0	13.8%	0.055
With stage 4 chronic kidney disease	0	1.5%	0.54
Diabetic polyneuropathy	0	24.6%	**0.00** **7**
Purine metabolism disturbances	12.5%	23.1%	0.27
Among them, gout	0	13.8%	0.055
Lipid and carbohydrate metabolism disorders			
MetS	71.4%	65.6%	0.62
Body mass index (BMI)	34.57 [29.48–40.04]	31.37 [27.88–34.31]	0.08
Obesity (BMI > 30)	71.4%	65.6%	0.62
Plasma triglycerides > 1.7 mmol/L	33.3%	50.8%	0.14
High-density lipoprotein < 0.9 mmol/L	4.2%	6.2%	0.72
Low-density lipoprotein > 4.5 mmol/L	8.3%	6.2%	0.72
Glucose, mmol/L	6.75 [6.39–6.89]	7.57 [6.89–10.15]	**0.00008**
C-peptide, nmol/L	1.13 [0.87–1.35]	1.02 [0.73–1.60]	0.82
HbA1c, %	5.8 [5.4–6.1]	6.6 [6.1–7.7]	**0.000002**
HOMA-IR (CP), Me [Q1-Q3]	4.00 [3.60–4.61]	4.54 [3.49–6.43]	0.12
HOMA-IR (CP) > 3.5	78.9%	72.9%	0.67
HOMA-islet, Me [Q1–Q3]	139.8 [119.6–178.9]	112.0 [90.3–138.1]	**0.004**
HOMA-islet < 167.58	87.9%	70%	0.06
TyG, Me [Q1–Q3]	8.97 [8.75–9.30]	9.33 [9.01–9.92]	**0.02**
TyG > 4.65	100%	100%	>0.05
Antidiabetic treatment of patients			
Biguanides	75%	92.3%	**0.028**
Sulfonylureas	0	15.4%	**0.041**
Sodium-glucose cotransporter-2 (SGLT2) inhibitors (gliflozins)	33.3%	70.8%	**0.0013**
Dipeptidyl peptidase-4 (DPP-4) inhibitors (gliptins) and glucagon-like peptide 1 (GLP-1) receptor agonists (glutides)	25%	53.8%	**0.015**
Insulin	0	6.2%	0.21

Note. NYHA—New York Heart Association; T2DM—type 2 diabetes mellitus; MetS—metabolic syndrome; BMI—body mass index; HbA1c—glycated hemoglobin; HOMA—homeostasis model assessment; TyG—triglyceride-glucose index; SGLT2—sodium-glucose cotransporter-2; DPP-4—dipeptidyl peptidase-4; GLP-1—glucagon-like peptide 1.

**Table 2:** Descriptive statistics of researched parameters in the groups.

Parameters		T2DM (*n* = 65)	Prediabetes (*n* = 24)	Control (*n* = 89)
Age, years	Me [Q1–Q3]	66 [62–73] ^C^	63 [52–71] ^C^	32 [26–43]
Gender	male/female, %	55.4/44.6	38.1/61.9	55/45
CRP, mg/L	Me [Q1–Q3]	4.63 [2.58–10.01] *^C^	3.00 [1.30–6.44] *	2.55 [0.94–4.51]
	3–10 mg/L, %	46.2	33.3	44.9
	>N (10 mg/L), %	26.2 ^C^	12.5 ^C^	0
IL-6, pg/mL	Me [Q1–Q3]	1.92 [0.86–4.49] *^C^	0.56 [0.00–1.94] *	0.80 [0.45–1.37]
	>N (5 pg/mL), %	21.5 ^C^	8.3	1.1
IL-8, pg/mL	Me [Q1–Q3]	12.37 [6.68–34.55] ^C^	8.34 [5.27–27.82] ^C^	1.73 [1.35–2.48]
	>N (10 pg/mL),%	58.5 ^C^	45.8 ^C^	0
IL-10, pg/mL	Me [Q1–Q3]	1.07 [0.09–2.74] *	0.22 [0.00–1.04] *	0.57 [0.00–1.95]
	>N (10 pg/mL), %	1.5	0	1.1
TNF-α, pg/mL	Me [Q1–Q3]	10.85 [0.00–51.54] ^C^	0.00 [0.00–42.72] ^C^	0.00 [0.00–0.36]
	>N (8 pg/mL), %	53.8 ^C^	37.5 ^C^	0
D-dimer, ng/mL	Me [Q1–Q3]	124.40 [80.15–288.73] *^C^	66.55 [46.42–123.26] *^C^	11.54 [4.94–30.28]
	100–250 ng/mL, %	40.0 ^C^	33.3 ^C^	5.6
	>N (250 ng/mL), %	27.7 *^C^	0.0 *	0
	>500 ng/mL, %	10.8 ^C^	0.0	0
Troponin I, ng/mL	Me [Q1–Q3]	0.00 [0.00–0.01] ^C^	0.00 [0.00–0.00]	0.00 [0.00–0.00]
	>0.2 ng/mL, %	7.7 ^C^	4.2	0
Myoglobin, ng/mL	Me [Q1–Q3]	12.79 [8.47–18.61] *^C^	9.76 [6.81–13.34] *	7.74 [5.61–13.32]
	>N (25 ng/mL) %	10.8	0	3.4
	>60 ng/mL %	0	0	0
Cortisol, nmol/L	Me [Q1–Q3]	420.3 [327.9–568.4] *^C^	317.3 [261.1–453.8] *	358.3 [278.2–450.1]
	>N (690 nmol/L), %	12.3 ^C^	8.3	3.4
	<N (100 nmol/L), %	3.1	0	0
PCT, ng/mL	Me [Q1–Q3]	0.05 [0.04–0.07] *^C^	0.03 [0.02–0.05] *^C^	0.03 [0.02–0.03]
	>N (0.1 ng/mL), %	13.8 ^C^	8.3	1.1
RL scale, point	Me [Q1–Q3]	1 [0–2] ^C^	0 [0–1] ^C^	0
	RL-0, %	40 ^C^	62.5 ^C^	98.9
	RL-1, %	27.7 ^C^	16.7 ^C^	1.1
	RL-2, %	23.1 ^C^	12.4 ^C^	0
	RL-3, %	7.7 ^C^	4.2	0
	RL-4, %	1.5	4.2	0
ChSI, point	Me [Q1–Q3]	1 [0–2] ^C^	0 [0–2] ^C^	0
LGSI, point	Me [Q1–Q3]	2 [1–2] *	1 [0–1] *	not defined
SMI/ChSI	%	18.5 ^C^	8.3 ^C^	0
hyper-ChSI	%	1.5	4.2	0
SMI/LGSI	%	56.9 ^C^	45.8 ^C^	0
SMI (in total)	%	75.4 ^C^	54.1 ^C^	0

Note. *—statistically significant differences between T2DM и Prediabetes groups, ^C^—statistically significant differences from the control group; RL—reactivity level; IL—interleukin; TNF—tumor necrosis factor; CRP—C-reactive protein; PCT—procalcitonin; ChSI—chronic systemic inflammation; LGSI—low-grade systemic inflammation; SMI—systemic meta-inflammation; T2DM—type 2 diabetes mellitus.

**Table 3:** Comparative analysis of complex immune–inflammatory indices in the groups.

Parameters		T2DM (*n =* 26)	Prediabetes (*n =* 11)	Control (*n =* 22)
Age, years	Me [Q1–Q3]	69 [62–73]	52 [48–65]	53 [49–60]
SII	Me [Q1–Q3]	365.5 [192.0–605.6]	200.3 [135.6–414.0] ^C^	387.9 [295.2–474.9] ^2^
	>660, %	19.2	9.1	9.1
NLR	Me [Q1–Q3]	1.62 [1.38–2.19] ^2^	1.01 [0.81–1.42] ^1,C^	1.56 [1.25–1.76] ^2^
	>4, %	0	0	0
PLR	Me [Q1–Q3]	84.4 [65.2–111.3] ^C^	90.9 [60.0–133.1] ^C^	113.0 [93.9–147.1] ^1,2^
	>165, %	3.8	9.1	18.2

Note. ^1^—statistically significant differences from T2DM; ^2^—statistically significant differences from prediabetes; ^C^—statistically significant differences from the control group; NLR—neutrophil-to-lymphocyte ratio; PLR—platelet-to-lymphocyte ratio; SII—systemic immune–inflammation index; T2DM—type 2 diabetes mellitus.

**Table 4:** Comparative analysis of an integrated group of patients with IR (prediabetes + T2DM) and a comparable age group of volunteers without IR according to the ChSI criteria.

Parameters	With IR	Without IR
*n*	89	22
Age (min–max)	42–83	61–83
Age, M ± Std. Dev.	65.2 ± 9.2	68.6 ± 5.9
Male/female, %	51.2/48.8	59.1/40.9
TNF-α > N (8 pg/mL), %	51.2	9.1 *
IL-6 > N (5 pg/mL), %	18.0	4.5
IL-8 > N (10 pg/mL), %	55.1	31.8
IL-10 > N (5/10 pg/mL ^1^),%	1.1	0
CRP 3–10 mg/L, %	42.7	45.5
CRP > N (10 mg/L), %	22.5	9.1
Troponin I > 0.2 ng/mL, %	6.7	0
Myoglobin > 60 ng/mL, %	0	0
Cortisol > 690 nmol/L or <100 nmol/L, %	13.5	0
D-dimer > N (250 ng/mL), %	20.2	0*
D-dimer > 500 ng/mL, %	7.9	0
RL-0%	46.1	81.8 *
RL-1%	24.7	18.2
RL ≥ 2%	29.2	0 *
ChSI, point, M ± Std. Dev.	1.22 ± 1.24	0.18 ± 0.39 *
ChSI %	15.7	0 *
LGSI ^2^, %	53.9	18.2

Note. In the group of patients with IR, the study was conducted using the ELISA method; in the group without IR, the study was conducted using the chemiluminescent immunoassay method (Immulite). In addition to the parameters of the ChSI scale, the table presents data on CRP 3–10 mg/L and D-dimer > 250 ng/mL. 1: <5 pg/mL is the normal value for IL-10 by the chemiluminescent immunoassay method, and <10 pg/mL is the normal value for ELISA. 2: in patients with IR, metabolic mechanisms play a leading role in the development of LGSI; the development of LGSI in the absence of IR is caused by inflamm-aging processes. *, *p* < 0.05.

**Table 5:** Area under the ROC curve (AUC ± standard error) for the studied parameters in the prognosis of insulin resistance.

Parameters	AUC ± St. Error	*p*-Level
C-reactive protein	0.692 ± 0.039	**<0.0001**
IL-6	0.631 ± 0.045	**0.003**
IL-8	0.957 ± 0.015	**<0.0001**
TNFα	0.727 ± 0.039	**<0.0001**
IL-10	0.542 ± 0.043	0.338
D-dimer	0.924 ± 0.02	**<0.0001**
Troponin I	0.605 ± 0.043	**0.015**
Myoglobin	0.656 ± 0.042	**<0.0001**
Cortisol	0.575 ± 0.043	0.085
Reactivity level (RL)	0.766 ± 0.037	**<0.0001**
ChSI scale	0.815 ± 0.034	**<0.0001**

Note. Statistically significant differences between groups are highlighted in bold.

**Table 6:** Area under the ROC curve (AUC ± standard error) for the studied parameters in the prognosis of T2DM (when examining patients diagnosed with type 2 diabetes and prediabetes).

Parameters	AUC ± St. Error	*p*-Level
C-reactive protein	0.657 ± 0.067	**0.024**
IL-6	0.675 ± 0.067	**0.012**
IL-8	0.578 ± 0.072	0.263
TNFα	0.593 ± 0.073	0.180
IL-10	0.664 ± 0.063	**0.018**
D-dimer	0.761± 0.006	**0.002**
Troponin I	0.527 ± 0.068	0.698
Myoglobin	0.656 ± 0.064	**0.025**
Cortisol	0.678 ± 0.068	**0.010**
Reactivity level (RL)	0.605 ± 0.069	0.128
ChSI scale	0.636 ± 0.068	**0.049**
HOMA-IR	0.618 ± 0.066	0.118
HOMA-islet	0.719 ± 0.062	**0.004**
TyG	0.675 ± 0.062	**0.022**

Note. Statistically significant differences between groups are highlighted in bold.

**Table 7:** Classification of chronic diseases based on the probability of detecting signs of ChSI.

Cluster	No.	Nosologies	SIR Detection (RL ≥ 1), %	ChSI Detection, %	RL ≥ 4, %
Cluster 1 (≤1% of ChSI)	1	Menopausal syndrome and essential hypertension, stages I-II	6.3	0	0
	2	Autoimmune thyroiditis	20.7	0	0
	3	Pelvic inflammatory disease	25.0	0	0
	4	Tuberculosis spondylitis	29.2	0	0
	5	Normal function of renal allograft	41.7	0	0
Cluster 2 (1.1–10% of ChSI)	6	Chronic heart failure, NYHA classes II-III	46.9	2.0	0
	7	Lung tuberculosis	20.0	6.0	0
	8	Psoriatic arthritis	66.7	8.3	0
	9	Prediabetes	37.5	8.3	4.2
	10	Chronic nonhealing wounds	81.0	9.5	0
Cluster 3 (10.1–50% of ChSI)	11	Ankylosing spondylitis	55.5	11.1	0
	12	Chronic rheumatic valvular heart disease	46.5	13.3	0
	13	Type 2 diabetes mellitus	60. 0	18.5	1.5
	14	Reactive arthritis	53.3	20.0	0
	15	Rheumatoid arthritis	69.0	31.0	0
	17	Chronic renal allograft dysfunction	91.3	43.5	0
Cluster 4 (>50% of ChSI)	18	Chronic limb threatening ischaemia	94.7	57.9	0
	19	Systemic lupus erythematosus	91.8	75.5	38.8
	20	End-stage renal disease	95.2	88.1	2.4
	21	Primary antiphospholipid syndrome	100	100	0

Note. The data are taken from previously conducted and published studies [21,47].

**Table 8:** Comparative analysis of parameters included in the RL scale and the ChSI scale in three control groups.

Parameters	Enzyme immunoassay (Lazurite)	Chemiluminescent Immunoassay (Immulite)	
No. of groups	1	2	3
Age range	18–55 years	18–55 years	61–83 years
*n*	89	50	22
Male/female, %	55/45	52/48	59/41
Age, years (M ± Std. Dev.)	33.8 ± 10.7	34.1 ± 10.4	68.5 ± 5.9
Age, years (Me [Q1–Q3])	32 [26–43]	33 [27–41]	68 [64–72]
TNF-α > N (8 pg/mL), %	0	2.0	9.1
IL-6 > N (5 pg/mL), %	1.1	0	9.1
IL-8 > N (10 pg/mL), %	0	2.0	31.8
IL-10 > N (5/10 pg/mL ^1^), %	1.1	0	0
CRP 3–10 mg/L, %	44.9	24.0	45.5
CRP > N (10 mg/L), %	0	0	9.1
Troponin I > 0.2 ng/mL, %	0	0	0
Myoglobin > N (25 ng/mL), %	3.4	2.0	31.8
Myoglobin > 60 ng/mL, %	0	0	0
Cortisol > N (690 nmol/L), %	3.4	0	0
Cortisol < N (138 nmol/L), %	0	0	0
D-dimer > N (250 ng/mL), %	0	0	0
RL-0, %	98.9	100	81.8
RL-1 (SIR criterion) ^2^, %	1.1	0	18.2
ChSI, %	0	0	0

Note. 1. The norm values are different for different methods (10 pg/mL for enzyme immunoassay; 5 pg/mL for chemiluminescent immunoassay). 2. RL-1 is confirmed by exceeding 2 SIR criteria (out of 5) by up to 2 times or by more significant changes in 1 criterion (see Table S4 in the Supplement). N—norm values.

**Table 9:** Chronic systemic inflammation (ChSI) scale.

ChSI Phenomena	Partial ChSI Criteria	Unit	Norm	ChSI Scale Points
Systemic inflammatory response	RL scale	Points (0 to 5)	0	1 RL point = 1 ChSI scale point
Microthrombosis	D-dimers > 500	ng/mL	<250	1 point
Systemic alteration	Myoglobin > 60	ng/mL	<25	1 point
	Troponin I > 0.2	ng/mL	<0.1	
Disfunction of the hypothalamic–pituitary–adrenal system	Cortisol > 690 Cortisol < 100	nmol/L	138–690	1 point

**Table 10:** The scale of low-grade systemic inflammation (LGSI).

LGSI Parameters	Values	Scale Points
CRP level	3–10 mg/L ^1^	1
	>10 mg/L	2
D-dimers level	250–500 ng/mL	1
	>500 ng/mL	2
Levels of pro-inflammatory cytokines: IL-6 (>5 pg/mL), IL-8 (>10 pg/mL), and TNFα (>8 pg/mL)	Elevated level of 1 cytokine	1
	Elevated levels of 2–3 cytokines	2
Cortisol level	>690 nmol/L	1
Presence of LGSI		≥2 points ^1^

Note. 1—in the presence of insulin resistance.

## Data Availability

The datasets analyzed during the current study are available from the corresponding author on reasonable request, as they contain information on the gender, age, and diagnosis of the patients.

## Supplementary Materials

The following supporting information can be downloaded at: https://www.mdpi.com/article/10.3390/ijms27198891/s1.

## Abbreviations

The following abbreviations are used in this manuscript:

ADA	American Diabetes Association
AUC	area under the curve
BMI	body mass index
CHF	chronic heart failure
ChSI	chronic systemic inflammation
CRP	C-reactive protein
DIC	disseminated intravascular coagulation
DPP -4	dipeptidyl peptidase-4
ESRD	end-stage renal disease
GLP-1	glucagon-like peptide 1
GLUT-4	glucose transporter type 4
HbA1c	glycated hemoglobin
HDL-C	high-density lipoprotein cholesterol
HIV	human immunodeficiency virus
HOMA	Homeostasis Model Assessment
HPA axis	hypothalamic–pituitary–adrenal axis
IL	interleukin
LGSI	low-grade systemic inflammation
MASLD	metabolically associated steatosis liver disease
Me	median
MetS	metabolic syndrome
NAFLD	nonalcoholic fatty liver disease
NYHA	New York Heart Association
NLR	neutrophil-to-lymphocyte ratio
PCT	procalcitonin
PLR	platelet-to-lymphocyte ratio
RL	reactivity level
RNA	ribonucleic acid
SGLT2	sodium-glucose cotransporter-2
SI	systemic inflammation
SII	systemic immune–inflammation index
SIR	systemic inflammatory response
SLE	systemic lupus erythematosus
SMI	systemic meta-inflammation
T2DM	type 2 diabetes mellitus
TnI	troponin I
TNF	tumor necrosis factor
TyG	triglyceride-glucose index
