# Supplementary material for: Meta-Inflammation in Type 2 Diabetes Mellitus: Theoretical, Methodological, and Clinical Aspects
Source: Int J Mol Sci. 2026 Oct 7;27(19):8891. doi: 10.3390/ijms27198891 (PMC13665223; doi:10.3390/ijms27198891)
Supplement: Supplementary file 1 [file ijms-27-08891-s001.zip › ijms-4603574-supplementary.pdf]

Supplementary Materials

Table S1. Comparative analysis of systemic inflammation parameters in patients depending on their COVID-19 status.

Parameters		Patients with a confirmed COVID-19 diagnosis (*n=32*)	Patients with no history of confirmed COVID-19 cases (*n=51*)
Age, years	Me [Q1-Q3]	67 [61-74]	66 [62-71]
Gender	male / female, %	65.6 / 34.4	42.3 / 57.7
CRP, mg/l	Me [Q1-Q3]	4.34 [2.20-10.22]	4.42 [2.07-9.46]
	3-10 mg/l, %	37.5	49.0
	> N (10 mg/l), %	28.1	19.6
IL-6, pg/ml	Me [Q1-Q3]	1.91 [0.91-6.40] *	1.60 [0.16-3.17] *
	> N (5 pg/ml), %	28.1	13.7
IL-8, pg/ml	Me [Q1-Q3]	16.77 [7.71-52.99]	11.81 [6.21-31.42]
	> N (10 pg/ml),%	59.4	56.9
IL-10, pg/ ml	Me [Q1-Q3]	0.73 [0.09-2.40]	0.94 [0.00-2.74]
	> N (10 pg/ml), %	0	2.0
TNF-α, pg/ ml	Me [Q1-Q3]	10.68 [0.00-82.38]	3.32 [0.00-31.61]
	> N (8 pg/ ml), %	56.3	49.0
D-dimer, ng/ml	Me [Q1-Q3]	108.95 [65.44-143.85]	154.03 [70.04-311.65]
	250 - 500 ng/ml, %	9.4	15.7
	>500 ng/ml, %	0.0 *	13.7 *
Troponin I, ng/ml	Me [Q1-Q3]	0.00 [0.00-0.01]	0.01 [0.00-0.01]
	>0.2 ng/ml, %	6.3	7.8
Myoglobin, ng/ml	Me [Q1-Q3]	12.71 [7.68-20.39]	10.64 [7.81-16.32]
	> N (25 ng/ml) %	9.4	7.8
	>60 ng/ml %	0	0
Cortisol, nmol/l	Me [Q1-Q3]	385.9 [296.3-554.7]	426.6 [321.2-568.4]
	> N (690 nmol/l), %	12.5	11.8
	< N (100 nmol/l), %	0	3.9
PCT, ng/ml	Me [Q1-Q3]	0.05 [0.03-0.07]	0.04 [0.03-0.06]
	> N (0.1 ng/ml), %	15.6	11.8
RL scale, point	Me [Q1-Q3]	1 [0-2]	1 [0-2]
	RL-0, %	37.5	47.1
	RL-1, %	25.0	27.5
	RL-2, %	21.9	19.6
	RL-3, %	12.5	3.9
	RL-4, %	3.1	2.0
ChSI, point	Me [Q1-Q3]	1 [0-2]	1 [0-2]
LGSI, point	Me [Q1-Q3]	2 [1-3]	1 [0-1]
ChSI	%	18.8	15.7
LGSI	%	37.5	39.2
Systemic meta-inflammation	%	56.3	54.9

Table S2. Comparative analysis of systemic inflammation parameters depending on the presence of metabolic syndrome in patients.

Parameters		with MetS (*n=54*)	without MetS (*n=25*)
Age, years	Me [Q1-Q3]	66 [61-71]	70 [51-74]
Gender	male / female, %	55.6 / 44.4	52.0 / 48.0
CRP, mg/l	Me [Q1-Q3]	4.98 [2.54-9.95]	3.80 [1.40-8.11]
	3-10 mg/l, %	46.3	40.0
	> N (10 mg/l), %	24.1	20.0
IL-6, pg/ml	Me [Q1-Q3]	1.69 [0.65-4.24]	1.97 [0.71-6.01]
	> N (5 pg/ml), %	16.7	28.0
IL-8, pg/ml	Me [Q1-Q3]	11.32 [6.40-45.44]	15.49 [6.22-34.30]
	> N (10 pg/ml),%	53.7	64.0
IL-10, pg/ ml	Me [Q1-Q3]	0.90 [0.07-2.48]	0.72 [0.00-2.74]
	> N (10 pg/ml), %	1.9	0
TNF-α, pg/ ml	Me [Q1-Q3]	10.48 [0.00-58.09]	3.32 [0.00-42.00]
	> N (8 pg/ ml), %	51.9	48.0
D-dimer, ng/ml	Me [Q1-Q3]	115.61 [65.84-219.54]	107.95 [70.14-165.40]
	250 - 500 ng/ml, %	14.5	8.0
	>500 ng/ml, %	7.3	12.0
Troponin I, ng/ml	Me [Q1-Q3]	0.00 [0.00-0.00] *	0.01 [0.00-0.06] *
	>0.2 ng/ml, %	5.6	12.0
Myoglobin, ng/ml	Me [Q1-Q3]	12.50 [8.03-18.04]	9.23 [7.51-16.59]
	> N (25 ng/ml) %	7.4	12.0
	>60 ng/ml %	0	0
Cortisol, nmol/l	Me [Q1-Q3]	435.2 [300.0-595.7]	365.9 [313.9-559.1]
	> N (690 nmol/l), %	11.1	16.0
	< N (100 nmol/l), %	0	8.0
PCT, ng/ml	Me [Q1-Q3]	0.05 [0.03-0.06]	0.04 [0.02-0.07]
	> N (0.1 ng/ml), %	14.8	12.0
RL scale, point	Me [Q1-Q3]	1 [0-2]	1 [0-2]
	RL-0, %	48.1	36.0
	RL-1, %	16.7	36.0
	RL-2, %	25.9	16.0
	RL-3, %	7.4	8.0
	RL-4, %	1.9	4.0
ChSI, point	Me [Q1-Q3]	1 [0-2]	1 [1-2]
LGSI, point	Me [Q1-Q3]	2 [1-3] *	1 [0-1] *
ChSI	%	14.8	24.0
LGSI	%	40.7	28.0
Systemic meta-inflammation	%	55.5	52.0

Table S3. Correlations in an integrated group of patients with carbohydrate metabolism disorders (T2DM+prediabetes), *n=89*.

Variable	CRP	IL-6	IL-8	TNFα	IL-10	D-dim	Tro	Myo	Cor	PCT	RL	ChSI point	Glucose	HbA1c	C-peptide	HOMA-B	HOMA-IR	HOMA-islet	TyG	BMI	age	LGSI point
CRP	1.00	0.42			0.53					0.21	0.24	0.23	0.25	0.30					0.23	0.23		0.58
IL-6	0.42	1.00	0.47	0.44	0.40	0.22				0.38	0.56	0.55										0.49
IL-8		0.47	1.00	0.92	0.32						0.89	0.82					0.23					0.60
TNFα		0.44	0.92	1.00	0.32					0.22	0.91	0.82							0.24			0.57
IL-10	0.53	0.40	0.32	0.32	1.00	0.25			0.22	0.37	0.35	0.36					0.27					0.56
D-dim		0.22			0.25	1.00															0.29	0.35
Tro							1.00							0.24								
Myo								1.00													0.28	
Cor					0.22				1.00	0.31			0.23									
PCT	0.21	0.38		0.22	0.40				0.31	1.00	0.25		0.29	0.26	0.24		0.40					0.27
RL	0.24	0.56	0.89	0.91	0.35					0.25	1.00	0.92										0.58
ChSI point	0.23	0.55	0.82	0.82	0.36						0.92	1.00										0.56
Glucose	0.25								0.23	0.29			1.00	0.78		-0.54	0.51	-0.54	0.63			
HbA1c	0.30						0.24			0.26			0.78	1.00		-0.65		-0.65	0.38			
C-peptide										0.24					1.00	0.69	0.86	0.69	0.36	0.25	-0.30	
HOMA-B													-0.54	-0.65	0.69	1.00	0.34					
HOMA-IR			0.23		0.27					0.40			0.51		0.86	0.34	1.00	0.34	0.59			
HOMA-islet													-0.54	-0.65	0.69		0.34	1.00				
TyG	0.23			0.24									0.63	0.38	0.36		0.59		1.00			
BMI	0.23														0.26					1.00		
age						0.30		0.28							-0.30				-0.22		1.00	0.29
LGSI point	0.58	0.49	0.60	0.57	0.56	0.35				0.27	0.58	0.56									0.29	1.00

Note. The table shows only statistically significant correlations.

Table S4. The ranges of biomarker concentrations and their corresponding RLs.

Marker	Normal	Ranges of absolute parameter values and corresponding RL					
		1	2	3	4	5	6
IL-6, pg/ml	<5.0	5-10	10-40	40-200	200-1000	>1000	-
IL-8, pg/ml	<10.0	10-25	25-100	100-500	500-2500	>2500	-
IL-10, pg/ml	<5.0	-	5-10	10-25	25-100	100-500	>500
TNFα, pg/ml	<8.0	8-16	16-40	40-160	160-800	>800	-
CRP, mg/dl	<1.0	1-3	3-15	>15	-	-	-

Note. The mean of three maximal RL values for each factor gave the RL (1 to 5) for each patient, which was used for further calculation of the SI score. RL – reactivity level; IL – interleukin; TNF – tumor necrosis factor; CRP – C-reactive protein. It is reasonable to determine the RL by the degree (multiplicity) to which it exceeds the maximum permissible values of the norm. For the method we use, the normal limits are given in the Normal column, and the ranges for determining RL are given in absolute values (pg/ml and mg/ml).

Table S5. Interpretation of reactivity levels (RLs) as a criterion for SIR in various forms of inflammation [21; 22; 58; 64].

RL	Canonical inflammation	Acute SI	ChSI/LGSI	Hyper-ChSI
**0**	Excludes SIR	Excludes acute SI	Verifies systemic meta-inflammation in presence of IR+CRP 3-10 mg/l + ≥1 additional criteria	Excludes hyper-ChSI
**1**	Typical severity of SIR for acute and, in some cases, chronic inflammation	Excludes acute SI	Verifies systemic meta-inflammation in presence of IR; it is characteristic of ChSI	Verifies hyper-ChSI if ≥2 additional criteria are present
**2**	It is typical for acute phlegmons and other variants of purulent acute inflammation, rarely for chronic inflammation without ChSI criteria	Verifies the depressive phase if 3 additional criterion are present	It is characteristic only of some marginal manifestations of the LGSI	It is typical for hyper-ChSI, verifies hyper-ChSI if ≥1 additional criteria are present
**3**	It is borderline variant of SIR in acute inflammation, not typical for chronic inflammation without ChSI	Verifies the depressive phase if 2 additional criteria are present	Excludes ChSI and LGSI	Verifies hyper-ChSI even without additional criteria
**4**	It is not characteristic of either acute or chronic canonical inflammation	The cytokine storm phase is confirmed if 1 additional criterion is present	Excludes ChSI and LGSI	Rare ChSI variants mimicking a "cytokine storm"
**5**	It is not characteristic of either acute or chronic canonical inflammation	Verifies the cytokine storm phase even without additional criteria	Excludes ChSI and LGSI	It is detected in approximately 2% of cases of systemic lupus erythematosus

Note. ChSI – chronic systemic inflammation, CRP – C-reactive protein, IR – insulin resistance, LGSI – low-grade systemic inflammation, RL – reactivity level, SI – systemic inflammation, SIR – systemic inflammatory response.
